# A reduction from an LWE problem to maximum independent set problems

**DOI:** 10.1038/s41598-023-34366-7

**Published:** 2023-05-02

**Authors:** Yasuhito Kawano

**Affiliations:** Center for Mathematical Science and Artificial Intelligence, College of Science and Engineering, Chubu University, 1200 Matsumoto-cho, Kasugai-shi, Aichi-Prefecture 487-0017 Japan

**Keywords:** Engineering, Mathematics and computing

## Abstract

The learning with errors (LWE) problem is a problem derived from machine learning that is believed to be intractable for quantum computers. This paper proposes a method that can reduce an LWE problem to a set of maximum independent set (MIS) problems, which are graph problems that are suitable for a quantum annealing machine to solve. The reduction algorithm can reduce an *n*-dimensional LWE problem to several small MIS problems with at most $2^{O(\sqrt{n})}$ nodes when the lattice-reduction algorithm used in the LWE-reduction method successfully finds short vectors. The algorithm is useful for solving LWE problems in a quantum-classical hybrid manner by using an existing quantum algorithm to solve the MIS problems. For example, the smallest LWE challenge problem is reduced to MIS problems with about 40,000 vertices. This result means that the smallest LWE challenge problem will be within the scope of a real quantum computer in the near future.

## Introduction

Verifying a quantum advantage (or supremacy) compared to a classical computer is one of the most exciting challenges of developing a quantum computer. In 2019, a quantum advantage was confirmed using a superconductor quantum device and the task of sampling the output of a pseudo-random quantum circuit^1^. The task was performed in 200 seconds by a newly developed 53-qubit quantum processor called “Sycamore,” whereas it was estimated that a state-of-the-art classical computer would take approximately 10,000 years to perform an equivalent task. In 2020, a quantum advantage was confirmed using a photonic quantum device and the task of boson sampling^2^. The sampling rate of the quantum device was faster than the sampling rate obtained using a state-of-the-art simulation strategy and supercomputers by a factor of $\sim 10^{14}$. Quantum advantages will be verified using quantum devices and intractable problems typified by sampling problems in the years to come.

There are three main approaches to verifying a quantum advantage. The first is to calculate an inversion of a classically hard one-way function, such as by performing factoring using Shor’s algorithm^3^. The second is to sample from a classically hard-to-sample distribution, such as by performing boson sampling^4^. The third is to verify that an untrusted device is truly quantum by using interactive protocols^5^.

In this paper, the problem of learning with errors (LWE)^6^ is studied as a candidate problem for verifying a quantum advantage; it belongs to the first approach. The LWE problem arises from machine learning and is believed to be intractable for a quantum computer. The intractability of the LWE problem is the basis for the security of LWE cryptography.

Using LWE problems to verify a quantum advantage has several advantages. First, a quantum-classical hybrid algorithm that solves LWE problems can be applied to machine learning. Second, the results of estimating the power of quantum computers can be applied to determine secure key lengths for LWE cryptography. Third, if an open LWE challenge problem was solved by a quantum computer, no one would be able to question the verification of the quantum advantage. (A classical simulator for pseudo-random quantum circuits as fast as Sycamore was proposed by Liu et al.^7^; hence, the proof of the quantum advantage^1^ has been questioned.) On the other hand, a disadvantage of this approach is that it is difficult to develop a quantum algorithm that solves LWE problems faster than classical algorithms. Estimating the number of qubits is also difficult. A naive encoding strategy requires a tremendously large number of qubits to encode an open LWE challenge problem. However, using the power of a classical computer can lessen the burden on the quantum (or quantum-inspired) computer.

To make it easier to solve an LWE problem using a quantum (or quantum-inspired) computer, we introduce a reduction of an LWE problem to another problem that is similar to an LWE problem. The reduction algorithm is called *LWE-reduction* in this paper. LWE-reduction can change the modulus of an LWE problem. It is easy to change the modulus to a larger number, whereas it is difficult to change the modulus to a smaller number in general. To change the modulus to a smaller number, a lattice-reduction algorithm is used. It is not guaranteed that an LWE problem can always be reduced to a modulo-two LWE-like problem in a practical length of time, since a lattice-reduction algorithm requires an exponential amount of time. However, a modulo-two LWE-like problem is much easier to work with than an LWE problem with a prime modulus larger than two. The reduced modulo-two LWE-like problem is made solvable by a quantum (or quantum-inspired) computer by converting it into a maximum independent set (MIS) problem^8^. It is known that an MIS problem can be encoded as a quadratic unconstrained binary optimization (QUBO) problem. Many quantum algorithms for MIS problems have been proposed^8,9^.

We calculated the upper bounds of the graphs of the MIS problems obtained by reducing an LWE problem using the LWE-reduction algorithm under the assumption that the lattice-reduction algorithm used in the LWE-reduction algorithm finds short vectors. For example, a 40-dimensional LWE problem can be reduced to MIS problems with graphs with tens of thousands of vertices and small weights ($-1$ or 2), where 40 is the smallest dimension of the LWE challenge problems^10^. Since $-1$ and 2 can be encoded in two bits, the minimum number of qubits necessary to solve a 40-dimensional LWE problem is estimated to be several tens of thousands. The MIS problems will lie within the scope of quantum computers in the near future. We implemented the LWE-reduction method and confirmed that a 40-dimensional LWE problem with a relative error of 0.005, which is the smallest challenge problem in the TU Darmstadt Learning With Errors Challenge^10^, can be reduced to an MIS problem with a graph of about 40,000 vertices.

### Related work

Many algorithms for LWE problems have been proposed. In particular, classical algorithms that solve LWE problems have been thoroughly studied.

The BKW algorithm was originally proposed by Blum, Kalai, and Wasserman^11^ as an algorithm for solving the learning parity with noise (LPN) problem, which is a subproblem of LWE. Later, the BKW algorithm was adapted to solve the LWE problem by Albrecht et al.^12^. The algorithm was further improved by Duc et al.^13^ and Kirchner et al.^14^.

Key recovery for LWE was proposed by Laine and Lauter^15^. They generalized the Boneh–Venkatesan method to a higher-dimensional generalization of the hidden number problem and used it for a key recovery attack on LWE that runs in polynomial time using the Lenstra–Lenstra–Lavász (LLL) lattice-reduction algorithm^16^ and Babai’s nearest plane algorithm^17^.

Bounded distance decoding (BDD) is a problem similar to the closest vector problem (CVP). The BDD approach for LWE is to reduce an LWE problem to a BDD problem and to search for the solution by pruning the search tree^18^. Another approach for LWE is to reduce the BDD problem to a unique shortest vector problem (uSVP) and to solve such instances using Kannan’s embedding approach^19^.

Quantum algorithms for solving the LWE problem have also been studied.

Göpfert, Vredendaal, and Wunderer^20^ proposed a quantum algorithm based on Howgrave–Graham’s classical hybrid attack. We call their algorithm the GVW algorithm. The GVW algorithm combines lattice-based techniques, such as lattice-reduction algorithms^16,21,22^, with (an improved) Grover’s search algorithm^23^. On the other hand, our algorithm combines lattice-based techniques, such as lattice-reduction algorithms, with optimization techniques using quantum annealing machines. The biggest difference between our algorithm and the GVW algorithm is that our algorithm reduces an LWE problem to several small MIS (graph) problems in a classical subroutine, whereas the GVW algorithm reduces an LWE problem to a BDD (lattice) problem and does not reduce it to graph problems.

Lv et al.^24^ proposed applying the quantum approximation optimization algorithm (QAOA)^25^ and variational quantum eigensolver (VQE)^26^ to solve LWE problems. The first algorithm reduces an LWE problem to a BDD problem and uses the QAOA to improve Babai’s nearest plane algorithm. The second algorithm reduces an LWE problem to a uSVP and solves the uSVP using a VQE. These algorithms work on noisy intermediate-scale quantum (NISQ) devices. Lv et al. carried out small-scale experiments and confirmed that their algorithms improve the quality of the solutions. The difference between these algorithms and our algorithm is that after reducing an LWE problem to a BDD problem, our algorithm further reduces it to several small MIS problems.

### Contributions of this paper

The main contribution of this paper is to estimate the smallest number of qubits necessary for verifying a quantum advantage using the LWE problem. As far as the author knows, such a number has not yet been estimated. Another contribution is to provide a quantum-inspired algorithm, called LWE-reduction, that reduces an LWE problem to another problem similar to LWE with a different modulus. This reduction is very useful for decreasing the required number of qubits.

## Preliminaries

In this section, the relevant background material is explained. The details are given in Refs.^27–30^.

### Learning with errors (LWE)

Let *n* be a positive integer. Let *q* be an integer larger than one. (*n* and *q* are called the dimension and the modulus of the LWE problem, respectively.) Let ${\mathbb Z}_q$ be ${\mathbb Z}/q{\mathbb Z}$. Let $\sigma >0$ be the standard deviation. Let $N(0,\sigma ^2)$ be the Gaussian distribution on ${\mathbb Z}_q$ with a mean value of zero and a standard deviation $\sigma$. For a fixed unknown vector $\mathbf{{s}}\in {\mathbb Z}_q^n$, let ${\mathscr {S}}_{\mathbf{{s}},\sigma }$ be a set of a polynomial number of samples $(\mathbf{{a}},\langle \mathbf{{a}},\mathbf{{s}}\rangle +e\ \textrm{mod}\ q)$, where $\mathbf{{a}}\leftarrow ^U{\mathbb Z}_q^n$ and $e\leftarrow N(0,\sigma ^2)$ are randomly selected. Solving an LWE problem consists of finding $\mathbf{{s}}\in {\mathbb Z}_q^n$ from a given ${\mathscr {S}}_{\mathbf{{s}},\sigma }$.

Let *m* be the number of samples in ${\mathscr {S}}_{\mathbf{{s}},\sigma }$. Denote $\mathbf{{a}}$ and *e* of the *i*th sample $(i=1,\ldots ,m)$ by $\mathbf{{a}}_i$ and $e_i$, respectively. Let *A* be the $m\times n$ matrix made from the row vectors $\mathbf{{a}}_1,\mathbf{{a}}_2,\ldots ,\mathbf{{a}}_m$. Let $\mathbf{{e}}$ be the *m*-dimensional column vector made from $e_1,e_2,\ldots ,e_m$. Let $\mathbf{{t}}$ be the *m*-dimensional column vector made from $\langle \mathbf{{a}}_i,\mathbf{{s}}\rangle +e_i\ \textrm{mod}\ q$. An LWE problem is the problem of finding a vector $\mathbf{{s}}$ that satisfies

1 $$\begin{aligned} \mathbf{{t}}\equiv A\mathbf{{s}}+\mathbf{{e}}\mod q \end{aligned}$$

such that the distributions of the components of $\mathbf{{e}}$ follow the Gaussian distribution $N(0,\sigma ^2)$. The above LWE problem will be denoted by the four-tuple $\left( A,\mathbf{{t}},q,\sigma \right)$.

The problem of finding an $\mathbf{{s}}$ that satisfies Eq. (1), where the distributions of the components of $\mathbf{{e}}$ may have different standard deviations, will be referred to as an *LWE-like problem*. In addition, the LWE-like problem allows fractional numbers to be components of $\mathbf{{t}}$ and $\mathbf{{e}}$. (Instead of a Gaussian distribution on ${\mathbb Z}_q$, a Gaussian distribution on $\{(t_j+z)\mod q|z\in {\mathbb Z}\}$ is used in the definition.) An LWE-like problem will be denoted by a four-tuple $\left( A,\mathbf{{t}},q,\{\sigma _j\}_{j=1}^m\right)$.

### Bounded distance decoding (BDD)

One of the standard strategies that is used to solve an LWE problem is BDD^31^. BDD is a problem similar to the closest vector problem.

Let $\mathbf{{X}}$ be a set of *n* *m*-dimensional row vectors, i.e., $\mathbf{{X}}=\{\mathbf{{x}}_1,\ldots ,\mathbf{{x}}_n\}$ for *m*-dimensional row vectors $\mathbf{{x}}_1,\ldots ,\mathbf{{x}}_n$. For an integer *q*, define the lattice $\Lambda _q(X)$ as follows:

$$\begin{aligned} \Lambda _q(X):=\left\{ \mathbf{{x}}\in {\mathbb Z}^m|\exists \mathbf{{s}}\in {\mathbb Z}^n\ \mathrm{s.t.}\ \mathbf{{x}}\equiv \mathbf{{s}}{} \mathbf{{X}}\mod q\right\} . \end{aligned}$$

Then, $\Lambda _q(X)$ is the lattice generated by the vectors in $\mathbf{{X}}$ and $q\textbf{u}_1,\ldots ,q\textbf{u}_m$, where $\textbf{u}_1,\ldots ,\textbf{u}_m$ are *m*-dimensional unit vectors. A basis of $\Lambda _q(X)$ is efficiently obtained by calculating the Hermite decomposition of the $(m+n)\times m$ matrix $$\left( \begin{matrix}qI_m\\ \mathbf{{X}}\end{matrix}\right) $$, where $I_m$ is the $m\times m$ identity matrix.

The BDD problem is as follows. Suppose that there is a number $0<\mu \le \frac{1}{2}$ such that, for a lattice *L* and a target vector $\mathbf{{t}}$,

$$\begin{aligned} {\textrm{dist}}(\mathbf{{t}},L):=\min _{\textbf{v}\in L}\left\| \mathbf{{t}}-\textbf{v}\right\| <\mu \lambda _1(L) \end{aligned}$$

holds, where $\lambda _1(L)$ is the length of the shortest vector in *L*. The BDD problem involves, given a basis of *L*, finding the vector $\textbf{v}\in L$ closest to $\mathbf{{t}}$.

The BDD problem can be regarded as a problem similar to the closest vector problem. Hence, the nearest plane algorithm^17^ for the closest vector problem can be applied to solve the BDD problem.

### Dual lattice

The dual lattice $\Lambda ^\perp _q(X)$ of $\Lambda _q(X)$ is defined as follows:

$$\begin{aligned} \Lambda ^\perp _q(X):=\left\{ {{\mathbf{y}}}\in {\mathbb Z}^m|\langle {{\mathbf{x}}},{{\mathbf{y}}}\rangle \equiv 0\mod q\ \text {for all}\ {{\mathbf{x}}}\in \Lambda _q(X)\right\} . \end{aligned}$$

Consider the case in which *X* is the set of row vectors of ${}^t\!A$, where *A* is the matrix of the LWE problem described by Eq. (1). A basis of $\Lambda ^\perp _q({}^t\!A)$ is calculated as the right-lower $m\times m$ matrix of the transforming matrix of the Hermite decomposition of the $2m\times m$ matrix $$\left( \begin{matrix} qI_m\\ {{\mathbf{B}}}\end{matrix}\right) $$, where ${{\mathbf{ B}}}$ is a basis of $\Lambda _q({}^t\!A)$.

We consider a superposition of quantum states on the dual lattice $\Lambda ^\perp _q({}^t\!A)$:

$$\begin{aligned} \left| \psi _\textbf{0}\right\rangle :=\frac{1}{\sqrt{\left| {\mathscr {D}}\right| }} \sum _{{{\mathbf{x}}}\in {\mathscr {D}}}\left| \mathbf{{x}}\right\rangle , \end{aligned}$$

where ${\mathscr {D}}:=\Lambda ^\perp _q({}^t\!A)\cap [0,q)^{\otimes m}$. By using the rotation gates according to the target vector $\mathbf{{t}}$, the following quantum state can be efficiently made:

$$\begin{aligned} \left| \psi _\mathbf{{t}}\right\rangle :=\frac{1}{\sqrt{\left| {\mathscr {D}}\right| }} \sum _{\mathbf{{x}}\in {\mathscr {D}}}e^{2\pi i\langle \mathbf{{x}},\mathbf{{t}}\rangle /q}\left| \mathbf{{x}}\right\rangle . \end{aligned}$$

In this paper, the quantum state $\left| \psi _\mathbf{{t}}\right\rangle$ will be called a $\mathbf{{t}}$-rotated state.

Let ${\mathscr {R}}$ be a small region around the origin contained in $[0,q)^{\otimes m}$ such that $\Lambda ^\perp _q({}^t\!A)\cap {\mathscr {R}}\ne \emptyset$. The $\mathbf{{t}}$-rotated state on ${\mathscr {R}}$ is defined as

$$\begin{aligned} \left| \psi _{\mathbf{{t}},{\mathscr {R}}}\right\rangle :=\frac{1}{\sqrt{\left| \Lambda ^\perp _q({}^t\!A)\cap {\mathscr {R}}\right| }} \sum _{\mathbf{{x}}\in \Lambda ^\perp _q({}^t\!A)\cap {\mathscr {R}}}e^{2\pi i\langle \mathbf{{x}},\mathbf{{t}}\rangle /q}\left| \mathbf{{x}}\right\rangle . \end{aligned}$$

Let $\mathbf{{c}}$ be the closest vector of $\mathbf{{t}}$ in $\Lambda _q({}^t\!A)$. Let $\mathbf{{e}}$ be $\mathbf{{t}}-\mathbf{{c}}$. It is obvious that $\left| \psi _{\mathbf{{t}},{\mathscr {R}}}\right\rangle =\left| \psi _{\mathbf{{e}},{\mathscr {R}}}\right\rangle$. Hence, if $\mathbf{{t}}\in \Lambda _q({}^t\!A)$, then $\left| \psi _{\mathbf{{t}},{\mathscr {R}}}\right\rangle =\left| \psi _{\textbf{0},{\mathscr {R}}}\right\rangle$. If $\mathbf{{t}}$ is close to a lattice point in $\Lambda _q({}^t\!A)$, then $\left| \psi _{\mathbf{{t}},{\mathscr {R}}}\right\rangle \approx \left| \psi _{\textbf{0},{\mathscr {R}}}\right\rangle$ since $\langle \mathbf{{e}},\mathbf{{x}}\rangle \approx 0$ for any $\mathbf{{x}}\in \Lambda ^\perp _q({}^t\!A)\cap {\mathscr {R}}$. The closer $\mathbf{{t}}$ is to a lattice point in $\Lambda _q({}^t\!A)$, the closer $\left| \psi _{\mathbf{{t}},{\mathscr {R}}}\right\rangle$ is to $\left| \psi _{\textbf{0},{\mathscr {R}}}\right\rangle$.

### Notation

The following notation will be used in this paper. The symbol $A_{[a_1:a_2,b_1:b_2]}$ represents a submatrix of an $m\times n$ matrix *A* consisting of the rows from $a_1$ to $a_2$ (including both $a_1$ and $a_2$) and the columns from $b_1$ to $b_2$ (including both $b_1$ and $b_2$), where $1\le a_1<a_2\le m$ and $1\le b_1<b_2\le n$. The numbers of the first (or last) row (or column) can be eliminated. A negative number indicates the row (or column) that is that number of rows (or columns) from the end. Thus, $A_{[:a,:b]}$ represents the left-upper $a\times b$ submatrix of *A* and $B_{[-a:,-b:]}$ represents the right-lower $a\times b$ submatrix of *B*.

## LWE-reduction

We begin by explaining the idea of LWE-reduction. Then, LWE-reduction is described in Algorithm 1.

### Idea

As explained in the previous section, by comparing $\left| \psi _{\mathbf{{t}},{\mathscr {R}}}\right\rangle$ and $\left| \psi _{\textbf{0},{\mathscr {R}}}\right\rangle$, it can be determined whether $\mathbf{{t}}$ is close to a lattice point in $\Lambda _q({}^t\!A)$ or not. However, it is difficult to find points in $\Lambda ^\perp _q({}^t\!A)\cap {\mathscr {R}}$ in general. To make it easier to find such points, we consider a new lattice by adding (interpolating) new points to $\Lambda ^\perp _q\left( {}^t\!A\right)$.

Let $n'$ and $q'$ be integers larger than one. Select $\mathbf{{x}}_1$, $\ldots$, $\mathbf{{x}}_{n'}$ $\not \in$ $\Lambda ^\perp _q\left( {}^t\!A\right)$ such that $q'{} \mathbf{{x}}_1,\ldots ,q'{} \mathbf{{x}}_{n'}\in \Lambda ^\perp _q({}^t\!A)$. The points represented by $\mathbf{{x}}_1,\ldots ,\mathbf{{x}}_{n'}$ are called the *interpolated points*. Let *L* be the lattice generated by $\Lambda ^\perp _q\left( {}^t\!A\right) \cup \left\{ \mathbf{{x}}_1,\ldots ,\mathbf{{x}}_{n'}\right\}$, i.e.,

2 $$\begin{aligned} L:={\mathscr {L}}\left( \Lambda ^\perp _q\left( {}^t\!A\right) \cup \{\mathbf{{x}}_1,\ldots ,\mathbf{{x}}_{n'}\}\right) . \end{aligned}$$

Then, finding points in $L\cap {\mathscr {R}}$ may be easier than finding points in $\Lambda ^\perp _q({}^t\!A)\cap {\mathscr {R}}$. Select $\mathbf{{y}}_1,\ldots ,\mathbf{{y}}_{m'}\in L\cap {\mathscr {R}}$ such that $\{\mathbf{{y}}_1,\ldots ,\mathbf{{y}}_{m'}\}$ are linearly independent and call $\mathbf{{y}}_1,\ldots ,\mathbf{{y}}_{m'}$ the *sample points*. (Note that the coordinates of $\mathbf{{x}}_1,\ldots ,\mathbf{{x}}_{n'}$, $\mathbf{{y}}_1,\ldots ,\mathbf{{y}}_{m'}$ are not integers in general.)

Let $\mathbf{{c}}$ be the lattice point in $\Lambda _q({}^t\!A)$ closest to $\mathbf{{t}}$. Let $\mathbf{{e}}$ be $\mathbf{{t}}-\mathbf{{c}}$. Since $\mathbf{{t}}=\mathbf{{c}}+\mathbf{{e}}$,

3 $$\begin{aligned} \left( \begin{matrix}\langle \mathbf{{y}}_1,\mathbf{{t}}\rangle \\ \vdots \\ \langle \mathbf{{y}}_{m'},\mathbf{{t}}\rangle \end{matrix}\right) \equiv \left( \begin{matrix}\langle \mathbf{{y}}_1,\mathbf{{c}}\rangle \\ \vdots \\ \langle \mathbf{{y}}_{m'},\mathbf{{c}}\rangle \end{matrix}\right) + \left( \begin{matrix}\langle \mathbf{{y}}_1,\mathbf{{e}}\rangle \\ \vdots \\ \langle \mathbf{{y}}_{m'},\mathbf{{ e}}\rangle \end{matrix}\right) \mod q. \end{aligned}$$

Equation (3) has three terms.

Since $\mathbf{{y}}_1,\ldots ,\mathbf{{y}}_{m'}\in L$, they are written as linear combinations of $\{\mathbf{{x}}_1,\ldots ,\mathbf{{x}}_{n'}$, ${\bar{{\mathbf{b}}}}_{1},\ldots ,{\bar{{\mathbf{b}}}}_m\}$, where $\{\bar{{\mathbf{b}}}_1,\ldots ,{\bar{{\mathbf{b}}}}_m\}$ is a basis of $\Lambda ^\perp _q({}^t\!A)$. Since $\langle {\bar{{\mathbf{b}}}}_1,\mathbf{{c}}\rangle \equiv \cdots \equiv \langle {\bar{{\mathbf{b}}}}_m,\mathbf{{c}}\rangle \equiv 0\mod q$, $\langle \mathbf{{y}}_1,\mathbf{{c}}\rangle ,\ldots ,\langle \mathbf{{y}}_{m'},\mathbf{{c}}\rangle$ are equal to linear combinations of $\langle \mathbf{{x}}_1,\mathbf{{c}}\rangle ,\ldots ,\langle \mathbf{{x}}_{n'},\mathbf{{c}}\rangle$ modulo *q*. Hence, Eq. (3) can be written as

4 $$\begin{aligned} \left( \begin{matrix}\langle \mathbf{{y}}_1,\mathbf{{t}}\rangle \\ \vdots \\ \langle \mathbf{{y}}_{m'},\mathbf{{t}}\rangle \end{matrix}\right) \equiv A'\left( \begin{matrix}\langle \mathbf{{x}}_1,\mathbf{{c}}\rangle \\ \vdots \\ \langle \mathbf{{x}}_{n'},\mathbf{{c}}\rangle \end{matrix}\right) + \left( \begin{matrix}\langle \mathbf{{y}}_1,\mathbf{{e}}\rangle \\ \vdots \\ \langle \mathbf{{y}}_{m'},\mathbf{{e}}\rangle \end{matrix}\right) \mod q \end{aligned}$$

for some integral $m'\times n'$ matrix $A'$. It can be easily seen that $\langle \mathbf{{x}}_1,\mathbf{{c}}\rangle ,\ldots ,\langle \mathbf{{x}}_{n'},\mathbf{{c}}\rangle$ are $\frac{q}{q'}\times$integers. By applying $\frac{q'}{q}$ to all terms, Eq. (4) can be converted into

5 $$\begin{aligned} \frac{q'}{q}\left( \begin{matrix}\langle \mathbf{{y}}_1,\mathbf{{t}}\rangle \\ \vdots \\ \langle \mathbf{{y}}_{m'},\mathbf{{t}}\rangle \end{matrix}\right) \equiv A'\left( \frac{q'}{q}\left( \begin{matrix}\langle \mathbf{{x}}_1,\mathbf{{c}}\rangle \\ \vdots \\ \langle \mathbf{{x}}_{n'},\mathbf{{c}}\rangle \end{matrix}\right) \right) + \frac{q'}{q}\left( \begin{matrix}\langle \mathbf{{y}}_1,\mathbf{{e}}\rangle \\ \vdots \\ \langle \mathbf{{y}}_{m'},\mathbf{{e}}\rangle \end{matrix}\right) \mod q', \end{aligned}$$

where all components of the second term are integers. Since $\mathbf{{e}},\mathbf{{y}}_1,\ldots ,\mathbf{{y}}_{m'}$ are short vectors, the third term of Eq. (5) is a short vector.

Regard the first and third terms as the target and error vectors, respectively, of a new LWE-like problem. Then, Eq. (5) looks similar to an LWE problem.

### LWE-reduction algorithm

Algorithm 1 reduces an LWE problem to an LWE-like problem with an arbitrary modulus. Whether the reduced LWE-like problem has a unique solution or not depends on the set of sample points ${\bar{{\mathbf{X}}}}$ in the algorithm.

#### Algorithm 1

Input $(A,\mathbf{{t}},q,\sigma )$, an *n*-dimensional LWE problem with *m* samples and a standard deviation $\sigma$, i.e., for an $m\times n$ matrix *A*, *m*-dimensional column vector $\mathbf{{t}}$, and integer *q* (not necessarily prime), there are column vectors $\mathbf{{s}}={}^t(s_1,\ldots ,s_n)$ and $\mathbf{{e}}={}^t(e_1,\ldots ,e_m)$ such that

6 $$\begin{aligned} \mathbf{{t}}\equiv A\mathbf{{s}}+\mathbf{{e}}\mod q \end{aligned}$$

and each $e_j$ $(j=1,\ldots ,m)$ follows the Gaussian distribution $N(0,\sigma ^2)$.

Input $m'$ and $n'$, positive integers less than or equal to *m*. ($m'$ and $n'$ represent the number of samples and the dimension of the reduced LWE-like problem, respectively.)

Input $q'$, an integer larger than one. ($q'$ represents the modulus of the reduced LWE-like problem.)

Calculate an $m'\times n'$ matrix $A'$ and an $m'$-dimensional column vector $\mathbf{{t}}'$ as follows:

1. Select a basis of $\Lambda _q({}^t\!A)$. It is denoted by ${{\mathbf{ B}}}$.
2. Select a basis of $\Lambda _q^\perp ({}^t\!A)$ such that all components of the last $n'$ row vectors are *q* times integers. It is denoted by ${\bar{{\mathbf{ B}}}}$.
3. Let ${\bar{\text { B}}}'$ be the basis defined by replacing the last $n'$ row vectors of ${\bar{\text { B}}}$ with $\frac{1}{q'}{\bar{{\mathbf{b}}}}_{m-n'+1},\ldots ,\frac{1}{q'}{\bar{{\mathbf{b}}}}_m$, respectively, i.e.,
  7 $$\begin{aligned} {\bar{{\mathbf{ B}}}}'&:=\left( \begin{matrix} {\bar{{\mathbf{b}}}}_1\\ \vdots \\ {\bar{{\mathbf{b}}}}_{m-n'}\\ \frac{1}{q'}{\bar{{\mathbf{b}}}}_{m-n'+1}\\ \vdots \\ \frac{1}{q'}{\bar{{\mathbf{b}}}}_m \end{matrix} \right) \end{aligned}$$
  8 $$\begin{aligned}&=\left( \begin{matrix} {\bar{{\mathbf{ B}}}}_{[:m-n',:]}\\ \frac{1}{q'}{\bar{{\mathbf{ B}}}}_{[-n':,:]} \end{matrix} \right) . \end{aligned}$$
  Here, $\frac{1}{q'}{\bar{{\mathbf{b}}}}_{m-n'+1},\ldots ,\frac{1}{q'}{\bar{{\mathbf{b}}}}_m\not \in {\mathscr {L}}({\bar{{\mathbf{ B}}}})$. (Note that ${\bar{{\mathbf{ B}}}}'$ is a matrix whose entries are rational numbers.)
4. Let ${\bar{{\mathbf{X}}}}$ be a linearly independent set of vectors ${{\bar{{\mathbf{x}}}}}_1,\ldots ,{{\bar{{\mathbf{x}}}}}_{m'}$ close to the origin in ${\mathscr {L}}({\bar{{\mathbf{ B}}}}')\setminus \{\textbf{0}\}$. (It need not be a basis of ${\mathscr {L}}({\bar{{\mathbf{ B}}}}')$.)
5. Let $l_1,\ldots ,l_{m'}$ be the $\ell _2$-norms $\Vert {\bar{{\mathbf{x}}}}_1\Vert ,\ldots ,\Vert {\bar{{\mathbf{x}}}}_{m'}\Vert$ of the vectors ${\bar{{\mathbf{x}}}}_1,\ldots ,{\bar{{\mathbf{x}}}}_{m'}$. Let $\sigma '$ be $\frac{q'}{q}\sigma$.
6. Let *T* be a matrix such that
  9 $$\begin{aligned} {\bar{{\mathbf{X}}}}=T{\bar{{\mathbf{ B}}}}'. \end{aligned}$$
  Note that any component of *T* is an integer. (If ${\bar{{\mathbf{X}}}}$ is a basis, then *T* is the transformation matrix from ${\bar{{\mathbf{ B}}}}'$ to ${\bar{{\mathbf{X}}}}$.)
7. An integral matrix $A'$ and a column vector $\mathbf{{t}}'$ with rational components are defined by
  10 $$\begin{aligned} A'&:= T_{[:,-n':]}\mod q'\ \textrm{and} \end{aligned}$$
  11 $$\begin{aligned} \mathbf{{t}}'&:=\frac{q'}{q}{\bar{{\mathbf{X}}}}{} \mathbf{{t}}\mod q'. \end{aligned}$$
  Output $$\left( A',\mathbf{{t}}',q',\{\sigma 'l_i\}_{i=1}^{m'}\right) $$.

#### Theorem 1

Let $(A,\mathbf{{t}},q,\sigma )$ be an *n*-dimensional LWE problem with *m* samples and a standard deviation $\sigma$, i.e., there is an *n*-dimensional column vector $\mathbf{{s}}$ and an *m*-dimensional error vector $\mathbf{{e}}={}^t(e_1,\ldots ,e_m)$ such that

12 $$\begin{aligned} \mathbf{{t}}\equiv A\mathbf{{s}}+\mathbf{{e}}\mod q, \end{aligned}$$

where $e_j$ follows the Gaussian distribution $N(0,\sigma ^2)$. Let ${\bar{{\mathbf{X}}}}$ be the set of linearly independent vectors selected at Step 4 of Algorithm 1. Let $\left( A',\mathbf{{t}}',q',\{\sigma 'l_i\}_{i=1}^{m'}\right)$ be the output of Algorithm 1 for the inputs $(A,\mathbf{{t}},q,\sigma )$, $q'$, $m'$, and $n'$. Let $\mathbf{{s}}'$ and $\mathbf{{e}}'$ be the $n'$-dimensional column vector in ${\mathbb Z}_{q'}^{n'}$ and the $m'$-dimensional column vector in ${\mathbb Q}_{q'}^{m'}$ defined by

13 $$\begin{aligned} \mathbf{{s}}'&:=\left( \frac{1}{q}\bar{{\mathbf{ B}}}_{[-n':,:]}(\mathbf{{t}}-\mathbf{{e}})\right) \mod q'\ {\textrm{and}} \end{aligned}$$

14 $$ e_{i}^{\prime } : = \left\{ {\begin{array}{*{20}l}    {\frac{{q^{\prime } }}{q}\left\langle {\overline{{\mathbf{x}}} _{i} ,{\mathbf{e}}} \right\rangle \quad \bmod \,q^{\prime } } \hfill & {{\text{if}}\;\frac{{q^{\prime } }}{q}\left\langle {\overline{{\mathbf{x}}} _{i} ,{\mathbf{e}}} \right\rangle \quad \bmod \,q^{\prime }  < \frac{{q^{\prime } }}{2}} \hfill  \\    {q^{\prime }  - \left( {\frac{{q^{\prime } }}{q}\left\langle {\overline{{\mathbf{x}}} _{i} ,{\mathbf{e}}} \right\rangle \quad \bmod \,q^{\prime } } \right)} \hfill & {{\text{otherwise}}} \hfill  \\   \end{array} } \right., $$

where $e_i'$ $(i=1,\ldots ,m')$ is the *i*th component of $\mathbf{{e}}'$. (Hence, $\mathbf{{e}}'\equiv \frac{q'}{q}\left\langle {\bar{{\mathbf{X}}}},\mathbf{{e}}\right\rangle \mod q'$.) Then,

15 $$\begin{aligned} \mathbf{{t}}'\equiv A'{} \mathbf{{s}}'+\mathbf{{e}}'\mod q'. \end{aligned}$$

In addition, the error $e_i'$ follows the Gaussian distribution $N(0,\sigma '^2l_i^2)$. Hence, $\mathbf{{s}}'$ is a solution of the LWE-like problem $\left( A',\mathbf{{t}}',q',\{\sigma 'l_i\}_{i=1}^{m'}\right)$.

#### Proof

By Eq. (9),

16 $$\begin{aligned} {\bar{{\mathbf{X}}}}(\mathbf{{t}}-\mathbf{{e}})=T{\bar{{\mathbf{ B}}}}'(\mathbf{{t}}-\mathbf{{e}}). \end{aligned}$$

Since ${\bar{{\mathbf{b}}}}_i\in {\mathscr {L}}({\bar{{\mathbf{B}}}})$ for any $i\le m-n'$ and $\mathbf{{t}}-\mathbf{{e}}\in {\mathscr {L}}(\mathbf{{B}})$, we have $\langle {\bar{{\mathbf{b}}}}_i,\mathbf{{t}}-\mathbf{{e}}\rangle \equiv 0\mod q$ for any $i\le m-n'$. Hence, ${\bar{{\mathbf{ B}}}}'_{[:m-n',:]}(\mathbf{{t}}-\mathbf{{e}})\mod q=0$. In addition, ${\bar{{\mathbf{B}}}}'_{[-n':,:]}=\frac{1}{q'}{\bar{{\mathbf{B}}}}_{[-n':,:]}$ according to Eq. (8). The right-hand side of Eq. (16) is then

17 $$\begin{aligned} T{\bar{{\mathbf{B}}}}'({{\mathbf{t}}}-{{\mathbf{e}}})&=T\left( \begin{array}{l} {\bar{{\mathbf{B}}}}'_{[:m-n',:]}\\ {\bar{{\mathbf{B}}}}'_{[-n':,:]} \end{array}\right) ({{\mathbf{t}}}-{{\mathbf{e}}})\nonumber \\&\equiv T\left( \begin{array}{l} \textbf{0}\\ {\bar{{\mathbf{ B}}}}'_{[-n':,:]}({{\mathbf{t}}}-{{\mathbf{e}}}) \end{array}\right) \mod q\nonumber \\&\equiv T_{[:,-n':]}{\bar{{\mathbf{ B}}}}'_{[-n':,:]}({{\mathbf{t}}}-{{\mathbf{e}}})\mod q\nonumber \\&\equiv \frac{1}{q'}T_{[:,-n':]}{\bar{{\mathbf{B}}}}_{[-n':,:]}({{\mathbf{t}}}-{{\mathbf{e}}})\mod q. \end{aligned}$$

Combining Eqs. (16) and (17) implies that

18 $$\begin{aligned} {\bar{{\mathbf{X}}}}{} \mathbf{{t}}\equiv \frac{1}{q'}T_{[:,-n':]}\bar{\text { B}}_{[-n':,:]}(\mathbf{{t}}-\mathbf{{e}})+{\bar{{\mathbf{X}}}}{} \mathbf{{e}}\mod q. \end{aligned}$$

Change the modulus from *q* to $q'$. Then,

19 $$\begin{aligned} \frac{q'}{q}{\bar{{\mathbf{X}}}}{{\mathbf{t}}}\equiv T_{[:,-n':]}\left( \frac{1}{q}{\bar{{\mathbf{B}}}}_{[-n':,:]}({{\mathbf{t}}}-{{\mathbf{e}}})\right) +\frac{q'}{q}{\bar{{\mathbf{X}}}}{{\mathbf{e}}}\mod q'. \end{aligned}$$

The $A',\mathbf{{t}}',\mathbf{{s}}'$, and $\mathbf{{e}}'$ defined by Eqs. (10), (11), (13), and (14) satisfy Eq. (15).

Next, we prove that for each $i=1,\ldots ,m'$, $e_i'$ follows the Gaussian distribution $N(0,\sigma '^2l_i^2)$. The mean value is obviously zero. It will now be shown that the standard deviation is $\sigma 'l_i$. Using Eq. (14), an error vector $\mathbf{{e}}$ is mapped to a vector equivalent to $\frac{q'}{q}{\bar{{\mathbf{X}}}}{} \mathbf{{e}}\mod q'$ by the LWE-reduction algorithm. Here, each component of $\mathbf{{e}}$ follows the Gaussian distribution with a mean value of zero and a standard deviation $\sigma$. Let ${\bar{{\mathbf{x}}}}_i=(\bar{x}_{i,1},\ldots ,{\bar{x}}_{i,m'})$ be the *i*th vector of ${\bar{{\mathbf{X}}}}$. Then, $\Vert {\bar{{\mathbf{x}}}}_i\Vert =l_i$. Since all components of $\mathbf{{e}}$ are independent, the composite standard deviation of the *i*th component of ${\bar{{\mathbf{X}}}}{} \mathbf{{e}}$ is then $\sqrt{\bar{ x}_{i,1}^2\sigma ^2+\cdots +\bar{x}_{i,m'}^2\sigma ^2}$ $=\sigma \sqrt{\bar{x}_{i,1}^2+\cdots +\bar{x}_{i,m'}^2}$ $=\sigma l_i$. Hence, the standard deviation of the *i*th error is $\frac{q'}{q}\cdot \sigma l_i =\sigma 'l_i$. $\square$

In general, the uniqueness of the solution of the LWE-like problem $\left( A',\mathbf{{t}}',q',\{\sigma 'l_i\}_{i=1}^{m'}\right)$ is not guaranteed. It depends on the selection of $\mathbf{{ B}}$, $\bar{{\mathbf{ B}}}$, ${\bar{{\mathbf{X}}}}$, $m'$, and $n'$. We introduce the standard settings for these parameters.

#### Definition 1

The *standard setting* of the parameters in Algorithm 1 is defined as follows:

- $\mathbf{{ B}}$ and ${\bar{{\mathbf{B}}}}$ are row vectors of the Hermite normal forms of the bases of $\Lambda _q({}^t\!A)$ and $\Lambda _q^\perp ({}^t\!A)$, respectively.
- ${\bar{{\mathbf{X}}}}$ represents the row vectors of $$\left( \begin{matrix} {\bar{{\mathbf{B}}}}_{[:m-n,m-n:]}&{}{\bar{{\mathbf{ B}}}}_{[:m-n,-n:]}-\frac{q}{q'}\Big \lfloor \frac{q'}{q}{\bar{{\mathbf{ B}}}}_{[:m-n,-n:]}\Big \rceil \\ O_{n,m-n}&{}\frac{q}{q'}I_n\end{matrix}\right) $$.
- $$m'=m$$ and $$n'=n$$.

When *q* is a prime number, the Hermite normal form of a basis of $\Lambda _q({}^t\!A)$ takes the form $$\left( \begin{matrix}I_n&{}\mathbf{{ B}}^*\\ O_{m-n,n}&{}qI_{m-n}\end{matrix}\right) $$ for some $n\times (m-n)$ matrix $\mathbf{{ B}}^*$, and the Hermite normal form of a basis of $\Lambda _q^\perp ({}^t\!A)$ takes the form $$\left( \begin{matrix} I_{m-n}&{}\bar{{\mathbf{B}}}^*\\ O_{n,m-n}&{}qI_n\end{matrix}\right) $$ for some $(m-n)\times n$ matrix $\bar{{\mathbf{ B}}}^*$. Hence, $\mathbf{{ B}}$ and $\bar{{\mathbf{ B}}}$ are row vectors of those matrices. Then, ${\bar{{\mathbf{X}}}}$ represents the row vectors of $$\left( \begin{matrix}I_{m-n}&{}\bar{{\mathbf{ B}}}^*-\frac{q}{q'}\Big \lfloor \frac{q'}{q}\bar{{\mathbf{ B}}}^*\Big \rceil \\ O_{n,m-n}&{}\frac{q}{q'}I_n\end{matrix}\right) $$.

Proposition 1 says that both the standard deviations of the distributions of the error components and the length among the closest lattice points of BDD defined from the LWE-like problem are about $\frac{q'}{q}$ times those of the LWE problem when *q* is a prime number and $q'$ and *m* are sufficiently large. Hence, it is expected that the LWE-like problem has a unique solution when it is reduced by the LWE-reduction algorithm in the standard setting when *q* is a prime number and $q'$ and *m* are sufficiently large.

#### Proposition 1

Let $(A,\mathbf{{t}},q,\sigma )$, $(A',\mathbf{{t}}',q',\{\sigma 'l_i\}_{i=1}^{m'})$, $m,n,m'$, and $n'$ have the same meanings as in Theorem 1. Suppose that $\mathbf{{ B}}$, $\bar{{\mathbf{ B}}}$, ${\bar{{\mathbf{X}}}}$, $m'$, and $n'$ are in the standard setting. Suppose that *q* is prime.

1. The standard deviations of the LWE-like problem are less than or equal to $\sigma '\sqrt{1+n\left( \frac{q}{2q'}\right) ^2}$, which is close to $\sigma '$ when $q'$ is sufficiently greater than *q*. Hence, the standard deviation of the LWE-like problem is about $\frac{q'}{q}$ ($=\frac{\sigma '}{\sigma }$) times that of the LWE problem.
2. The length among the closest lattice points of BDD defined from $(A',\mathbf{{t}}',q')$ is about $\left( \frac{q'}{q}\right) ^{1-n/m}$ times that of $(A,\mathbf{{t}},q)$. It is close to $\frac{q'}{q}$ when *m* is sufficiently greater than *n*.

#### Proof

1. Since *q* is prime, ${\bar{{\mathbf{X}}}}$ is the set of row vectors of $$\left( \begin{matrix} I_{m-n}&{}{\bar{{\mathbf{B}}}}^*-\frac{q}{q'}\Big \lfloor \frac{q'}{q}{\bar{{\mathbf{ B}}}}^*\Big \rceil \\ O_{n,m-n}&{}\frac{q}{q'}I_n\end{matrix}\right) $$. Each component of ${\bar{{\mathbf{ B}}}}^*-\frac{q}{q'}\Big \lfloor \frac{q'}{q}{\bar{{\mathbf{ B}}}}^*\Big \rceil$ is less than or equal to $\frac{q}{2q'}$. Each vector of ${\bar{{\mathbf{X}}}}$ is then shorter than or equal to $\sqrt{1+n\left( \frac{q}{2q'}\right) ^2}$. By Theorem 1, the standard deviations are less than or equal to $\sigma '\sqrt{1+n\left( \frac{q}{2q'}\right) ^{2}}$.

2. Since $\Lambda _{q}({}^{{t}}{\!A})$ has the form $$\left( \begin{matrix} I_{n}&{}\mathbf{{B}}^{*}\\ O_{m-n,n}&{}qI_{m-n} \end{matrix}\right) $$, the Gaussian heuristic^30^, Page 344] of ${\Lambda }_{q({}^{t}{\!A})}$ is equal to $\sqrt{\frac{m}{2\pi e}}q^{\frac{m-n}{m}}$. Similarly, the Gaussian heuristic of ${\Lambda }_{{q{'}}}({}^{{t}}{A'})$ is equal to $\sqrt{\frac{m'}{2\pi e}}q'^{\frac{m'-n'}{m'}}$. Dividing the latter by the former yields $\left( \frac{q'}{q}\right) ^{1-n/m}$ since $m'=m$ and $n'=n$. $\square$

Suppose that *q* is prime and there is a unique solution of the LWE-like problem $(A',\mathbf{{t}}',q',\{\sigma 'l_i\}_{i=1}^{m'})$. In the standard setting, the solution of the LWE problem can be calculated from the solution of the LWE-like problem as follows.

#### Theorem 2

Let $(A,\mathbf{{t}},q,\sigma )$, $(A',\mathbf{{t}}',q',\{\sigma 'l_i\}_{i=1}^{m'})$, $m,n,m'$, and $n'$ have the same meanings as in Theorem 1. Suppose that $\mathbf{{ B}}$, $\bar{{\mathbf{ B}}}$, ${\bar{{\mathbf{X}}}}$, $m'$, and $n'$ are in the standard setting. Suppose that *q* is a prime number, $q'\ge 2q$, and there is a unique solution $\mathbf{{s}}'$ of the LWE-like problem $(A',\mathbf{{t}}',q',\{\sigma 'l_i\}_{i=1}^{m'})$. Then, the unique solution $\mathbf{{s}}$ of the LWE problem $(A,\mathbf{{t}},q,\sigma )$ can be efficiently calculated from $\mathbf{{s}}'$. (Note that $q'$ can be a composite number.)

#### Proof

Since ${\bar{{\mathbf{B}}}}_{[-n:,:]}$ is in the standard setting, ${\bar{{\mathbf{B}}}}_{[-n:,:]}$ is the $n\times m$ matrix $$\left( \begin{matrix}O_{n,m-n}&qI_n\end{matrix}\right) $$. Hence, $\frac{1}{q}{\bar{{\mathbf{B}}}}_{[-n:,:]}$ is the $n\times m$ matrix $$\left( \begin{matrix}O_{n,m-n}&I_n\end{matrix}\right) $$. This implies that $\frac{1}{q}{\bar{{\mathbf{B}}}}_{[-n:,:]}(\mathbf{{t}}-\mathbf{{e}})=\mathbf{{t}}-\mathbf{{e}}$. By Eq. (13),

20 $$\begin{aligned} \mathbf{{s}}'\equiv \mathbf{{t}}-\mathbf{{e}}\mod q'. \end{aligned}$$

Since $q'\ge 2q$ and all components of $\mathbf{{t}}$ are in [0, *q*), $\mathbf{{t}}$ and $\mathbf{{e}}$ are determined from Eq. (20). Hence, $\mathbf{{s}}$ can be efficiently calculated from Eq. (6). $\square$

## Quantum-classical hybrid algorithm

By using the LWE-reduction algorithm, an LWE problem with a modulus *q* can be reduced to an LWE-like problem with a modulus $q'$. As shown in the previous section, it is expected that the LWE-like problem will have a unique solution when *q* is a prime number and $q'$ and *m* are sufficiently large. However, when it comes to solving an LWE-like problem, a smaller $q'$ is better. Unfortunately, there are two problems with changing the modulus to a smaller number by using the LWE-reduction algorithm. One is that the uniqueness of the solution of the reduced LWE-like problem is not guaranteed. The other is that even if the solution of the LWE-like problem is unique, the solution of the LWE problem cannot be easily calculated from the solution of the LWE-like problem obtained from Algorithm 1 when $q'$ is coprime to *q*. In this section, we present a method for reducing an LWE problem to an LWE-like problem with a smaller modulus and solving that LWE problem.

The idea is to make the modulus of an LWE problem small in two stages, i.e., the modulus is increased to a large composite number in the first stage and then reduced to its divisor in the second stage. In addition, a lattice-reduction algorithm is used to find short vectors of ${\bar{{\mathbf{X}}}}$ in the second stage. For example, the modulus *q* is increased to $2^r$ for some $r\ge \log _2q$ in the first stage and is reduced to two in the second stage. The LWE problem with a prime modulus is then reduced to modulo-two LWE-like problems. We assume that the errors of the LWE-like problems are sufficiently small. By ignoring the errors, the modulo-two LWE-like problems can be represented as maximum satisfiability (MAX-SAT) problems. By solving these MAX-SAT problems, partial information about the solution of the LWE problem is obtained. To determine the solution, the LWE-reduction algorithm and an algorithm for MAX-SAT problems are run recursively. Since a MAX-SAT problem has been reduced to a maximum independent set (MIS) problem^32^ and a number of quantum algorithms for MIS problems are available^8,9^, the algorithm can be performed by quantum and classical computers in a hybrid manner.

Suppose that $(A,\mathbf{{t}},2^r,\sigma )$ is an LWE problem obtained using Algorithm 1 in the standard setting. (More precisely, it could have different standard deviations, according to Theorem 1; however, we assume that they are the same because Algorithm 1 in the standard setting outputs an LWE-like problem with an error vector whose components follow almost the same standard deviations. [See Proposition 1.]) The next algorithm (Algorithm 2) describes the second stage, which recursively calls Algorithm 1 with $q'=2,4,8,\ldots$, and reduces the LWE problem to modulo-two LWE-like problems (Eq. 22).

### Algorithm 2

Input $(A,\mathbf{{t}},2^r,\sigma )$, which is an *n*-dimensional LWE problem with *m* samples and a standard deviation $\sigma$, i.e., for an integral $m\times n$ matrix *A*, an *m*-dimensional column vector $\mathbf{{t}}$ with rational components, and an integer *r*, there are unique integral column vectors $\mathbf{{s}}={}^t(s_1,\ldots ,s_n)$ and rational $\mathbf{{e}}={}^t(e_1,\ldots ,e_m)$ such that

21 $$\begin{aligned} \mathbf{{t}}\equiv A\mathbf{{s}}+\mathbf{{e}}\mod 2^r, \end{aligned}$$

and each $e_j$ $(j=1,\ldots ,m)$ follows the Gaussian distribution $N(0,\sigma ^2)$.

Change the indices of the row vectors of *A* so that $A_{[-n:,:]}\mod 2$ is invertible in ${\mathbb Z}_2$. Denote the resulting matrix by the same symbol *A*.

For $i=0,1,\ldots ,r-1$, calculate the *n*-dimensional vector $\mathbf{{s}}_i$, whose components are bits, as follows. We start with $i=0$. Set $\mathbf{{t}}_0:=\mathbf{{t}}$ and $\sigma _0:=\sigma$.

1. Input $(A,\mathbf{{t}}_i,2^{r-i},\sigma _i)$, *m*, *n*, and 2 (as $m'$, $n'$, and $q'$, respectively) to Algorithm 1, which uses the Hermite normal form ${\bar{{\mathbf{B}}}}_i$ of a basis of $\Lambda _{2^{r-i}}^\perp ({}^t\!A)$ at Step 2. Let $(A'_i,\mathbf{{t}}'_i,2,\{\sigma '_il_{ij}\}_{j=1}^{m})$ be an output. Here, the $A'_i$’s are integral and the $\mathbf{{t}}'_i$’s have rational numbers as components.
2. Let $\mathbf{{s}}'_i$ be a vector whose components are bits that is a solution of the LWE-like problem $\left( A'_i,\mathbf{{t}}'_i,2,\{\sigma '_il_{ij}\}_{j=1}^{m}\right)$, i.e., $\mathbf{{s}}'_i$ satisfies
  22 $$\begin{aligned} \mathbf{{t}}'_i\equiv A'_i\mathbf{{s}}'_i+\mathbf{{e}}'_i\mod 2, \end{aligned}$$
  where the *j*th component of $\mathbf{{e}}'_i$ follows the Gaussian distribution $N\left( 0,\sigma _i'^2l_{ij}^2\right)$.
3. Calculate the *n*-dimensional vector $\mathbf{{s}}_i$, with bits as components, that satisfies
  23 $$\begin{aligned} \left( \frac{1}{2^{r-i}}{\bar{{\mathbf{B}}}}_{i[-n:,:]}(A \mathbf{{s}}_i\mod 2^{r-i})\right) \mod 2=\mathbf{{s}}'_i. \end{aligned}$$
4. If $i<r-1$, then set
  24 $$\begin{aligned} \mathbf{{t}}_{i+1}&:=\frac{1}{2^{i+1}}\left( \mathbf{{t}}-A\left( \sum _{j=0}^i2^j \mathbf{{s}}_j\right) \right) \ {\textrm{and}} \end{aligned}$$
  25 $$\begin{aligned} \sigma _{i+1}&:=\frac{1}{2^{i+1}}\sigma , \end{aligned}$$
  increment *i*, and go to the first step.
5. Output $$\sum _{j=0}^{r-1}2^j \mathbf{{s}}_j$$.

The output of Algorithm 2 is expected to be equal to the solution $\mathbf{{s}}$ of Eq. (21), as we will see below.

### Theorem 3

Let $(A,\mathbf{{t}},2^r,\sigma )$ be an *n*-dimensional LWE problem with *m* samples and standard deviations $\sigma$, i.e., there is a unique integral *n*-dimensional column vector $\mathbf{{s}}$ and a unique rational *m*-dimensional error vector $\mathbf{{e}}={}^t(e_1,\ldots ,e_m)$ such that

26 $$\begin{aligned} \mathbf{{t}}\equiv A\mathbf{{s}}+\mathbf{{e}}\mod 2^r, \end{aligned}$$

where $e_j$ $(j=1,\ldots ,m)$ follows the Gaussian distribution $N(0,\sigma ^2)$. Let the $A'_i$’s, $\mathbf{{t}}'_i$’s, $\mathbf{{s}}'_i$’s, and $\mathbf{{s}}_i$’s be the same as in Algorithm 2. It is assumed that for each $i=0,1,\ldots ,r-1$, the standard deviations $\{\sigma '_il_{ij}\}_{j=1}^{m}$ are sufficiently small, so that the LWE-like problem $\left( A'_i,\mathbf{{t}}'_i,2,\{\sigma _i'l_{ij}\}_{j=1}^{m}\right)$ has a unique solution. In addition, it is assumed that for all $i=0,1,\ldots ,r-1$, the components of ${\bar{{\mathbf{B}}}}_{i[-n:,:]}$ are even integers. Then, for the inputs $(A,\mathbf{{t}},2^r,\sigma )$, *m*, and *n*, the output of Algorithm 2 is equal to the solution $\mathbf{{s}}$ of the LWE problem $(A,\mathbf{{t}},2^r,\sigma )$.

### Proof

It is inductively shown that $\mathbf{{s}}_i$ is equal to the vector of bits consisting of the $(i+1)$st least significant bits of the components of $\mathbf{{s}}$.

For $i=0$, Theorem 1 holds. Set $\mathbf{{s}}_0$ to $\mathbf{{s}}$. Set $\mathbf{{t}}_0$ and $\mathbf{{e}}_0$ to $\mathbf{{t}}$ and $\mathbf{{e}}$, respectively. The solution $\mathbf{{s}}_0'$ of the LWE problem $(A'_0,\mathbf{{t}}'_0,2,\{\sigma '_0l_{0j}\}_{j=1}^{m})$ then satisfies Eq. (13), i.e.,

27 $$\begin{aligned} \left( \frac{1}{2^r}{\bar{{\mathbf{B}}}}_{0[-n:,:]}(A\mathbf{{s}}_0\mod 2^r)\right) \mod 2=\mathbf{{s}}'_0. \end{aligned}$$

Let ${\bar{{\mathbf{s}}}}_0$ be the vector obtained by changing the least significant bit of each component of $\mathbf{{s}}_0$ to zero. We want to show that

28 $$\begin{aligned} \left( \frac{1}{2^r}{\bar{{\mathbf{B}}}}_{0[-n:,:]}(A{\bar{{\mathbf{s}}}}_0\mod 2^r)\right) \mod 2=\textbf{0}. \end{aligned}$$

Since ${\bar{{\mathbf{B}}}}_0$ is a basis of $\Lambda _{2^r}^\perp ({}^t\!A)$, ${\bar{{\mathbf{B}}}}_{0[-n:,:]}A\equiv O_n\mod 2^r$. Since the components of ${\bar{{\mathbf{s}}}}_0$ are even, ${\bar{{\mathbf{B}}}}_{0[-n:,:]}A{\bar{{\mathbf{s}}}}_0\equiv \textbf{0}\mod 2^{r+1}$. Dividing both sides by $2^r$ yields $\frac{1}{2^r}{\bar{{\mathbf{B}}}}_{0[-n:,:]}A{\bar{{\mathbf{s}}}}_0\equiv \textbf{0}\mod 2$. By the assumption that the components of ${\bar{{\mathbf{B}}}}_{0[-n:,:]}$ are even, $\frac{1}{2^r}{\bar{{\mathbf{B}}}}_{0[-n:,:]}\cdot 2^r\equiv O_{n,m}\mod 2$. Then, $\frac{1}{2^r}{\bar{{\mathbf{B}}}}_{0[-n:,:]}(A{\bar{{\mathbf{s}}}}_0\mod 2^r)\equiv \textbf{0}\mod 2$, which yields Eq. (28). Equations (27) and (28) imply that

29 $$\begin{aligned} \left( \frac{1}{2^r}{\bar{{\mathbf{B}}}}_{0[-n:,:]}(A\left( \mathbf{{s}}_0-{\bar{{\mathbf{s}}}}_0\right) \mod 2^r)\right) \mod 2=\mathbf{{s}}'_0. \end{aligned}$$

Hence, the vector consisting of the least significant bit of each component of $\mathbf{{s}}_0$ is a solution of Eq. (23) for $i=0$. This vector is the only solution of Eq. (23) for $i=0$ because ${\bar{{\mathbf{ B}}}}_{0[-n:,-n:]}$ is an upper-triangular matrix (note that ${\bar{{\mathbf{ B}}}}_0$ is the Hermite normal form) and $A_{[-n:,:]}$ is invertible in ${\mathbb Z}_2$.

Suppose that for all $j<i$, $\mathbf{{s}}_j$ is equal to the vector of bits consisting of the $(j+1)$st least significant bits of the components of $\mathbf{{s}}$. Let $\mathbf{{s}}_i$ be $\frac{1}{2^i}\left( \mathbf{{s}}-\sum _{j=0}^{i-1}2^j \mathbf{{s}}_j\right)$. Let ${\bar{{\mathbf{s}}}}_i$ be the vector obtained by changing the least significant bit of the components of $\mathbf{{s}}_i$ to zero. By Eqs. (21) and (24),

30 $$\begin{aligned} \mathbf{{t}}_i\equiv A\mathbf{{s}}_i+\mathbf{{e}}_i\mod 2^{r-i}, \end{aligned}$$

where the *j*th component of $\mathbf{{e}}_i$ follows the Gaussian distribution $N(0,\frac{\sigma ^2}{2^{2i}})$. Hence, $\mathbf{{s}}_i$ is the solution of the LWE problem $(A,\mathbf{{t}}_i,2^{r-i},\sigma _i)$. Similar to the proof of Eq. (29), we can prove that

31 $$\begin{aligned} \left( \frac{1}{2^{r-i}}{\bar{{\mathbf{ B}}}}_{i[-n:,:]}(A\left( \mathbf{{s}}_i-{\bar{{\mathbf{ s}}}}_i\right) \mod 2^{r-i})\right) \mod 2=\mathbf{{s}}'_i. \end{aligned}$$

Hence, the vector consisting of the least significant bit of the components of $\mathbf{{s}}_i$ is a unique solution of Eq. (23) for *i*. $\square$

## Graph size and Qubit number

As shown in the previous section, an LWE problem can be reduced to modulo-two LWE-like problems (Step 2 of Algorithm 2). This section calculates an upper bound for the size of the graphs that result from converting one of these modulo-two LWE-like problems and estimates the number of qubits needed to solve an LWE problem using the quantum-classical hybrid algorithm under the assumption that for each $i=0,1,\ldots ,r-1$, the standard deviations $\{\sigma '_il_{ij}\}_{j=1}^{m}$ are sufficiently small, so that the LWE-like problem $\left( A'_i,\mathbf{{t}}'_i,2,\{\sigma '_il_{ij}\}_{j=1}^{m}\right)$ has a unique solution.

Suppose that there is a positive number $\delta$ such that all errors of the LWE-like problem $\left( A'_i,\mathbf{{t}}'_i,2,\{\sigma '_il_{ij}\}_{j=1}^{m}\right)$ are in the range $[-1+\delta ,1-\delta ]$. Let $\Omega _{i\delta }$ be the set $\{j|0\le t'_{ij}<\delta \vee 1-\delta<t'_{ij}<1+\delta \vee 2-\delta<t'_{ij}<2\}$, where $\mathbf{{t}}'_i={}^t(t'_{i1},\ldots ,t'_{im})$. Then,

32 $$\begin{aligned} \Big \lfloor t'_{ij}\Big \rceil \equiv A'_{i[j,:]}{} \mathbf{{s}}'_i\mod 2 \end{aligned}$$

for $j\in \Omega _{i\delta }$, where $\Big \lfloor t'_{ij}\Big \rceil$ is the rounding integer of $t'_{ij}$. If $m_\delta :=\#\Omega _{i\delta }\ge n$, then the solution of the MAX-SAT of the simultaneous equations of Eq. (32) ($j\in \Omega _{i\delta }$) satisfies all the equations of Eq. (22).

Actually, since we do not know that there is a positive number $\delta$ such that all components of the errors of the LWE-like problem $\left( A'_i,\mathbf{{t}}'_i,2,\{\sigma '_il_{ij}\}_{j=1}^{m}\right)$ are in the range $[-1+\delta ,1-\delta ]$, we will determine $\delta$ from a randomly generated LWE problem with the same dimension and relative error.

Reducing the simultaneous equations of Eq. (32) ($j\in \Omega _{i\delta }$) to a quadratic unconstrained binary optimization (QUBO) problem consists of two reductions, a reduction from Eq. (32) to a conjunctive normal form (CNF) and a reduction from a CNF to a QUBO. The second reduction is the reduction from a MAX-SAT problem of a CNF with a total of *t* literals to a maximum independent set (MIS) problem of a graph with *t* vertices, following Chapuis et al.^8^. Since the weights on the edges of the graph are coded in two bits (they are 2 and $-1$ in the paper), the number of qubits necessary to solve the QUBO that represents the MIS problem is a few times the number of vertices. The first reduction is studied below.

First, we evaluate the number of clauses and the number of literals of a CNF reduced from a parity equation.

### Lemma 1

A logical formula

33 $$\begin{aligned} b\leftrightarrow a_1\oplus a_2\oplus \cdots \oplus a_k \end{aligned}$$

can be expressed as a CNF with $2^k$ clauses and $(k+1)$ literals for each clause. (Hence, the total number of literals is $(k+1)2^k$.)

### Proof

Equation (33) is equal to

34 $$\begin{aligned} \lnot b\oplus a_1\oplus a_2\oplus \cdots \oplus a_k. \end{aligned}$$

Hence, it is sufficient to show that a *k*-variable $(\ge 2)$ logical formula

35 $$\begin{aligned} a_1\oplus a_2\oplus \cdots \oplus a_{k+1} \end{aligned}$$

can be expressed as a CNF with $2^k$ clauses and $(k+1)2^k$ literals.

Define $\psi _k$ and $\phi _k$ $(k\ge 1)$ by

36 $$\begin{aligned} \psi _1&\equiv a_1, \end{aligned}$$

37 $$\begin{aligned} \phi _1&\equiv a_1, \end{aligned}$$

38 $$\begin{aligned} \psi _{k+1}&\equiv (\psi _k\vee a_{k+1})\wedge (\lnot \phi _k\vee \lnot a_{k+1}),\ \textrm{and} \end{aligned}$$

39 $$\begin{aligned} \phi _{k+1}&\equiv (\phi _k\wedge \lnot a_{k+1})\vee (\lnot \psi _k\wedge a_{k+1}), \end{aligned}$$

where “mod 2” is omitted. (The term “mod 2” is omitted in similar contexts hereafter.) Then, for any $k\ge 1$,

40 $$\begin{aligned} \psi _k\leftrightarrow \phi _k\leftrightarrow a_1\oplus \cdots \oplus a_k \end{aligned}$$

because if $k=1$, then Eq. (40) holds by the definitions given in Eqs. (36) and (37), and if Eq. (40) holds for *k*, then

$$\begin{aligned} \psi _{k+1}&\equiv (\psi _k\vee a_{k+1})\wedge (\lnot \phi _k\vee \lnot a_{k+1})\\&\leftrightarrow a_1\oplus \cdots \oplus a_k\oplus a_{k+1} \ \textrm{and}\\ \phi _{k+1}&\equiv (\phi _k\wedge \lnot a_{k+1})\vee (\lnot \psi _k\wedge a_{k+1})\\&\leftrightarrow a_1\oplus \cdots \oplus a_k\oplus a_{k+1} \end{aligned}$$

hold.

Since Eq. (40) holds, it is sufficient to show that $\psi _k$ can be expressed as a CNF with $2^k$ clauses and $(k+1)2^k$ literals. It can be inductively proved that

- for any $k\ge 1$, $\psi _k$ is equal to a CNF with $2^{k-1}$ clauses and $k2^{k-1}$ literals, and
- for any $k\ge 1$, $\phi _k$ is equal to a DNF with $2^{k-1}$ clauses and $k2^{k-1}$ literals.

It is obvious from Eqs. (36) and (37) that (a) and (b) are true for $k=1$. If (a) and (b) are true for *k*, then

(c) $\psi _k$ and $\lnot \phi _k$ are equal to CNFs with $2^{k-1}$ clauses and $k2^{k-1}$ literals, and
(d) $\phi _k$ and $\lnot \psi _k$ are equal to DNFs with $2^{k-1}$ clauses and $k2^{k-1}$ literals.

Hence, (a) and (b) are true for $k+1$ by Eqs. (38) and (39). $\square$

### Theorem 4

Let $A=(a_{ij})_{1\le i\le m,1\le j\le n}$ be an $m\times n$ matrix of bits, and let $\mathbf{{t}}=(t_i)_{1\le i\le m}^T$ be an *m*-dimensional vector of bits. Let $\mathbf{{s}}={}^t(s_1,\ldots ,s_n)$ be a vector consisting of logical variables. The equation

41 $$\begin{aligned} \mathbf{{t}}\equiv A\mathbf{{s}}\mod 2 \end{aligned}$$

can be expressed as a CNF with at most $n+\mu (2^{\bar{ n}}-1)+\nu (2^{{\bar{n}}-1}-1)$ variables and at most

42 $$\begin{aligned} 12(\mu (2^{\bar{n}}-1)+\nu (2^{\bar{n}-1}-1))+m\Big \lceil \frac{n}{{\bar{n}}}\Big \rceil 2^{\Big \lceil \frac{n}{{\bar{n}}}\Big \rceil -1} \end{aligned}$$

total literals, where $\bar{n}:=\Big \lceil \sqrt{n}\Big \rceil$, $\mu :=n-(\bar{n}-1)\lceil \frac{n}{\bar{n}}\rceil$, and $\nu :=\bar{ n}\lceil \frac{n}{{\bar{n}}}\rceil -n$. An upper bound of the total number of literals is $2^{O(\sqrt{n})}$.

### Proof

It is easily checked that $n=\underbrace{\bar{n}+\cdots +\bar{n}}_\mu +\underbrace{(\bar{n}-1)+\cdots +(\bar{n}-1)}_\nu$. Since each component of *A* is a bit, each row of *A* has *n* bits. Divide each row of *A* into $\underbrace{\bar{n},\ldots ,\bar{n}}_\mu ,\underbrace{(\bar{n}-1),\ldots ,(\bar{n}-1)}_\nu$ bits. Then, each row of *A* can be represented as a set of $\mu$ $\bar{n}$-bit numbers and $\nu$ $(\bar{n}-1)$-bit numbers. Let *C* be the $m\times (\mu +\nu )$ matrix consisting of those numbers. Let $c_{i,j}$ be the (*i*, *j*)th component of *C*.

To express Eq. (41) in terms of $c_{i,j}$, new logical variables $\{x_1,\ldots ,x_n\}$ are introduced. Divide $\{x_1,\ldots ,x_n\}$ into $\mu +\nu$ sets, each of which has $\bar{n}$ or $\bar{n}-1$ components, as follows:

43 $$\begin{aligned}&\{x_1,\ldots ,x_{\bar{n}}\} \end{aligned}$$

44 $$\begin{aligned}&\quad \vdots \nonumber \\&\{x_{(\mu -1){\bar{n}}+1},\ldots ,x_{\mu \bar{n}}\} \end{aligned}$$

45 $$\begin{aligned}&\{x_{\mu \bar{n}+1},\ldots ,x_{\mu \bar{n}+(\bar{n}-1)}\}\end{aligned}$$

46 $$\begin{aligned}&\quad \vdots \nonumber \\&\{x_{\mu \bar{n}+(\nu -1)(\bar{n}-1)+1},\ldots ,x_{\mu \bar{n}+\nu (\bar{n}-1)}\}. \end{aligned}$$

In addition, for each set of Eqs. (43)–(44), the logical variables $y_{i,j}$ $(i=1,\ldots ,\mu ;j=1,\ldots ,2^{{\bar{n}}}-1)$ are introduced. For each set of Eqs. (45)–(46), the logical variables $y_{i,j}$ $(i=\mu +1,\ldots ,\mu +\nu ;j=1,\ldots ,2^{{\bar{n}}-1}-1)$ are introduced. The number of newly introduced logical variables $y_{i,j}$ is $\mu (2^{{\bar{n}}}-1)+\nu (2^{{\bar{n}}-1}-1)$. Since the number of logical variables $x_i$ is *n*, the total number of newly introduced logical variables is $n+\mu (2^{{\bar{n}}}-1)+\nu (2^{{\bar{n}}-1}-1)$.

Define the relation between $x_1,\ldots ,x_n$ and $y_{i,j}$ as follows:

47 $$\begin{aligned} y_{i,j}\leftrightarrow {\left\{ \begin{array}{ll} y_{i,j-{\textrm{Rest}}(j)}\oplus y_{i,{\textrm{Rest}}(j)}&{}\text {if Rest}(j)\ne 0,\\ x_{i{\bar{n}}-\log _2j}&{}\text {if Rest}(j)=0\ {\textrm{and}}\ i\le \mu ,\\ x_{\mu {\bar{n}}+(i-\mu )({\bar{n}}-1)-\log _2j}&{}\text {if Rest}(j)=0\ \textrm{and}\ i>\mu . \end{array}\right. } \end{aligned}$$

Here, $\textrm{Rest}(j)$ is the number obtained by eliminating the most significant bit of *j*. (For example, when *j* is 11, since the binary expression of *j* is $[1011]_2$, $\textrm{Rest}(j)=[11]_2=3$.) By Lemma 1, the total number of literals of the CNF equivalent to Eq. (47) is at most 12. Since the number of newly introduced logical variables $y_{i,j}$ is $\mu (2^{{\bar{n}}}-1)+\nu (2^{{\bar{n}}-1}-1)$, the total number of literals of the CNF equivalent to Eq. (47) is at most $12\left( \mu (2^{{\bar{n}}}-1)+\nu (2^{{\bar{n}}-1}-1)\right)$.

The *i*th equation of Eq. (41) can be written as follows:

48 $$\begin{aligned} {\left\{ \begin{array}{ll} y_{i,c_{i,1}}\oplus \cdots \oplus y_{i,c_{i,\mu }}\oplus y_{i,c_{i,\mu +1}}\oplus \cdots \oplus y_{i,c_{i,\mu +\nu }}&{}\text {if}\ b_i=1,\\ \lnot (y_{i,c_{i,1}}\oplus \cdots \oplus y_{i,c_{i,\mu }}\oplus y_{i,c_{i,\mu +1}}\oplus \cdots \oplus y_{i,c_{i,\mu +\nu }})&{}\text {if}\ b_i=0. \end{array}\right. } \end{aligned}$$

Here, $y_{i,c_{i,j}}$ is defined as an empty component when $c_{i,j}=0$. By Lemma 1, the total number of literals of the CNF equivalent to Eq. (48) is $(\mu +\nu )2^{\mu +\nu -1}$. Since the number of equations is *m*, the total number of literals of the CNF equivalent to Eq. (48) is at most $m(\mu +\nu )2^{\mu +\nu -1}$. Since $\mu +\nu =n-({\bar{n}}-1)\lceil \frac{n}{{\bar{n}}}\rceil +{\bar{n}}\lceil \frac{n}{{\bar{n}}}\rceil -n=\lceil \frac{n}{{\bar{n}}}\rceil$, the total number of literals is bounded by Eq. (42).

The total number of literals is then $2^{O(\sqrt{n})}$ since ${\bar{n}}$ and $\lceil \frac{n}{{\bar{n}}}\rceil$ are $O(\sqrt{n})$ and *m* is bounded by a polynomial in *n*. $\square$

**Figure 1:**
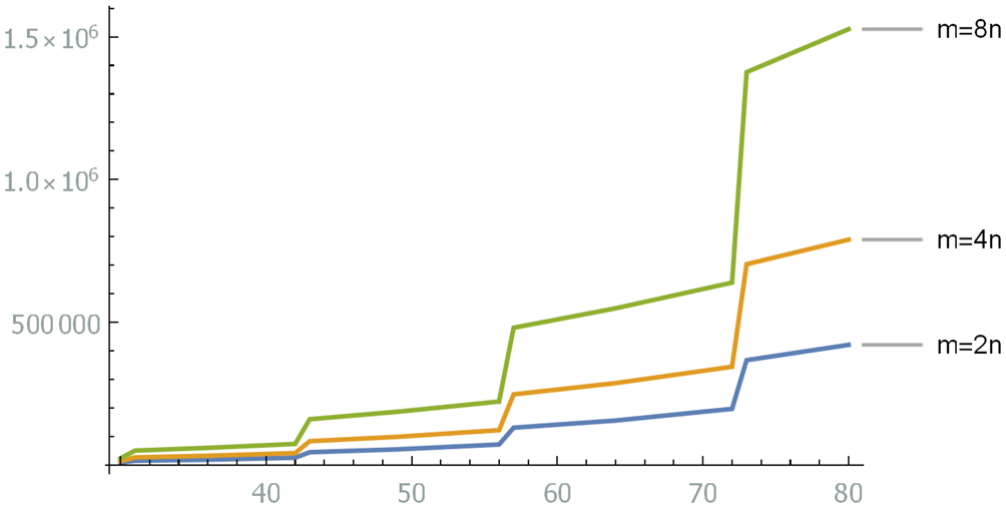
Upper bounds (Eq. 42) of the numbers of vertices of graphs reduced from *n*-dimensional LWE problems. They depend on the number of conditions $m_\delta$ in Eq. (32). The number of qubits needed to encode the reduced problem is a few times the upper bound. For example, when $n=40$ and $m_\delta =4n$, the graph shows that the upper bound is about 40,000. Hence, approximately a few times 40,000 qubits are required to encode the reduced problem.

The number of vertices in Eq. (42) depends on the number of conditions $m_\delta$ in Eq. (32). Figure 1 shows Eq. (42) for $m_\delta =2n,4n,8n$.

## Complexity analysis

We analyze the complexities of the proposed algorithms. Algorithm 1 in the standard setting efficiently reduces an LWE problem to an LWE-like problem. Proposition 2 states the time complexity of the algorithm.

### Proposition 2

If the standard setting is used, Algorithm 1 can be performed in polynomial time in terms of *m*, $\log _2p$, and $\log _2p'$.

### Proof

Steps 1 and 2 calculate the Hermite normal forms; this involves the most time-consuming computations in Algorithm 1. It is known that a Hermite normal form is computable in polynomial time. More precisely, both the number of algebraic operations and the number of binary digits of all intermediate numbers are bounded by polynomials of the length of the input data^33^. The sizes of the matrices in Steps 1 and 2 are $(m+n)\times m$ and $2m\times m$, respectively. The lengths of the binary digits of the components of those matrices are $O(\log _2p)$. Since $n\le m$, the time complexities of Steps 1 and 2 are polynomial in terms of *m* and $\log _2p$. The time complexities of the other steps are easy to calculate. (The $\ell _2$-norm calculation of Step 5 limits the decimal places to a finite depth since there is no need to calculate infinite digits for this application.) The lengths (denominators and numerators) of the components of ${\bar{{\mathbf{ B}}}}'$, ${\bar{{\mathbf{X}}}}$, *T*, $\textbf{A}'$, and $\mathbf{{t}}'$ are bounded by polynomials of $\log _2p$ and $\log _2p'$. Since $m=m'$ and $n=n'$ in the standard setting, Steps 3–7 of Algorithm 1 can be performed in polynomial time in terms of *m*, $\log _2p$, and $\log _2p'$. $\square$

On the other hand, Algorithm 2 is a heuristic algorithm. It is not guaranteed that the algorithm will output the correct answer for an input LWE problem even if sufficient time and space are provided. The reason for this is as follows.

To obtain the correct answer for an LWE problem using Algorithm 2, the solution of Eq. (22) must be successfully calculated in each loop. In our algorithm, instead of solving Eq. (22), an MIS problem obtained from Eq. (22) is solved. However, it is not guaranteed that the solution of Eq. (22) can be calculated from a solution of the MIS problem. This is because the solution of Eq. (22) may not satisfy Eq. (32) for all $j\in \Omega _{i\delta }$. The smaller the number of Eq. (32) that holds for the solution of Eq. (22), the more difficult it is for a quantum annealing machine to find the solution. To reduce the burden on the quantum annealing machine that solves the MIS problem, a lattice-reduction algorithm such as the Lenstra–Lenstra–Lavász (LLL) algorithm or BKZ algorithm^16,21,22^ is assumed to be used at Step 1 of Algorithm 2. Since vectors found by a lattice-reduction algorithm are set as sample points, we can expect the error (the third term) of Eq. (22) to be small. Unfortunately, a lattice-reduction algorithm takes a very long time to run. The LLL algorithm is a polynomial-time algorithm, but its performance is inadequate. The BKZ algorithm can find a shorter vector in general, but it needs a longer amount of time.

Even with such a shortcoming, it is worth considering using our algorithm to solve an LWE problem. The power of a quantum computer is definitely useful when solving an LWE problem. However, one of the biggest obstacles is that challenge problems are often too big to encode on today’s quantum computers. By reducing the number of qubits needed to encode an LWE problem, our algorithm makes it possible for a quantum computer to solve this problem.

## Implementation

We made a prototype of the LWE-reduction algorithm that reduces an LWE problem to MIS problems. The prototype was written in the Wolfram language (Mathematica^34^) and Python (SageMath^35^). The computer experiments were performed on an Ubuntu machine with two Intel(R) Xeon(R) E5-2680 v4 processors @ 2.40 GHz (each processor has 14 cores and 28 threads) and 256 GB of memory.

The TU Darmstadt Learning With Errors Challenge^10^ is used as the benchmark. The LWE challenge problems are sorted according to their dimensions and relative errors. The dimensions are $40,45,50,\ldots$ and the relative errors are $0.005,0.010,0.015,\ldots$. For each dimension and relative error pair, an LWE problem with these parameters is presented. The other parameters are set as follows: $m=n^2$ and *q* is the least prime number larger than *m*. The smallest problem is a problem with a dimension of 40 and a relative error of 0.005; it was solved by a classical computer in 2016. The problem with a dimension of 40 and a relative error of 0.035 was one of the unsolved problems as of June 21, 2021.

**Figure 2:**
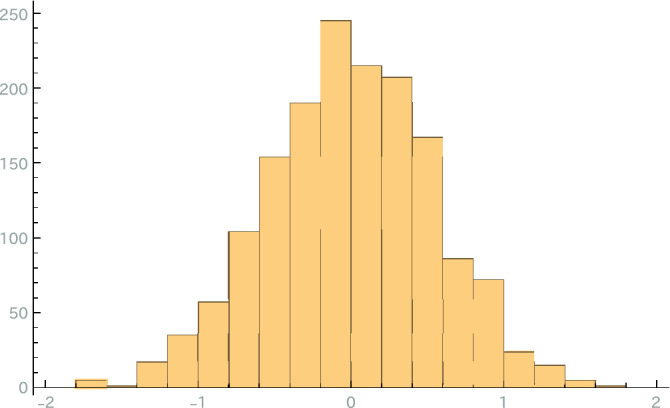
Histogram of the error distributions of an LWE-like problem $\left( A'_0,\mathbf{{t}}'_0,2,\{\sigma '_0l_{0j}\}_{j=1}^m\right)$ that was reduced from the LWE challenge problem with a dimension of 40 and a relative error of 0.005^10^. It is calculated from the solution shown on the site. The histogram shows that most of the components of the error are in the range $[-0.95,0.95]$; hence, $\delta$ is selected to be 0.05. $m_\delta$, which is the number of equations (conditions), is then calculated as 159; it is larger than the number of variables.

We explain how to use LWE-reduction to reduce the 40-dimensional LWE challenge problem with a relative error of 0.005 to a graph. To select $\delta >0$ (see the section “Graph Size and Qubit Number”), an LWE problem with the same dimension (40) and relative error (0.005) is randomly generated. (Hence, we know the solution and error.) Algorithm 2 calculates the LWE-like problems $\left( A'_i,\mathbf{{t}}'_i,2,\{\sigma '_il_{ij}\}_{j=1}^{m}\right)$. Since we know the solution and error of the LWE problem, we can calculate the errors of the LWE-like problems. For each *i*, the distribution of the components of the error is then similar to the distributions shown in Fig. 2.

**Figure 3:**
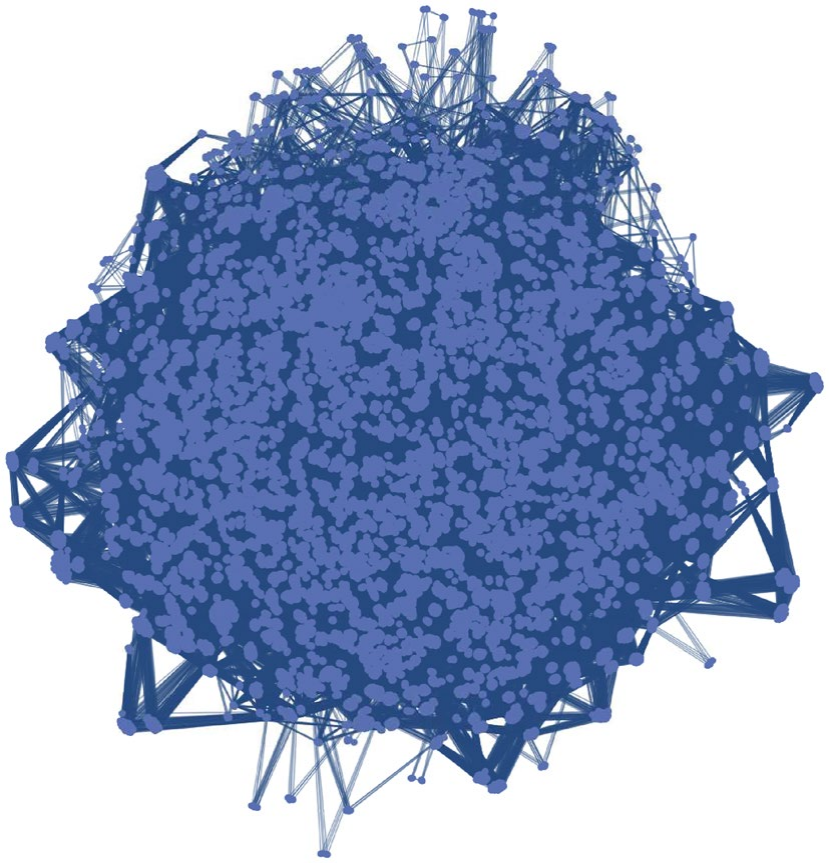
Graph of an MIS problem reduced from the TU Darmstadt LWE challenge problem with a dimension of 40 and a relative error of 0.005 using the reduction algorithm. The graph has 36,664 vertices and 1,982,176 edges.

This figure shows that most of the components of the error are in the range $[-0.95,0.95]$. Since LWE problems with the same dimension and relative error have the same difficulty level, most of the errors of the LWE-like problem that was reduced from the LWE challenge problem with $n=40$, $\alpha =0.005$, and $i=1$ are thought to be in the range $[-0.95,0.95]$. Let $\delta$ be 0.05. Calculate $m_{0.05}$ $(=\#\Omega _{0.05})$ for the LWE challenge problem and check if $m_{0.05}$ is larger than the dimension (40). (In this example, $m_{0.05}$ was 159.) The LWE-like problem $\left( A'_1,\mathbf{{t}}''_1,2,\{\sigma l_j\}_{j=1}^{m'}\right)$ is then reduced to a MAX-SAT problem that represents Eq. (32) for $j\in \Omega _{0.05}$. Figure 3 shows a graph of an MIS problem that was reduced from the MAX-SAT problem. The graph has 36,664 vertices and 1,982,176 edges.

We tried to reduce the LWE challenge problem with a dimension of 40 and a relative error of 0.035, which was still open as of June 21, 2021, to an MIS problem using the same strategy. An LWE problem with the same dimension and relative error was randomly generated and the error was calculated. Unfortunately, too many of the absolute values of the components of the error were larger than one. To make most of the error components smaller than one, it is necessary to find shorter vectors using a lattice-reduction algorithm. Replacing the lattice-reduction algorithm used in the prototype with a state-of-the-art algorithm would make the errors smaller.

## Conclusion

This paper proposed a reduction from an LWE problem to a set of maximum independent set (MIS) problems. The algorithm is useful for solving LWE problems in a quantum-classical hybrid manner by using an existing quantum algorithm for the MIS problems. There are several reasons to study quantum-classical hybrid algorithms for LWE problems. First, quantum-classical hybrid algorithms that solve LWE problems can be applied to machine learning. Second, the results of estimating the complexities of quantum-classical hybrid algorithms for LWE problems can be applied to determine secure key lengths for LWE cryptography. Third, if an open LWE challenge problem was solved by a quantum computer, no one would be able to question the verification of the quantum advantage. Successfully solving an open LWE challenge problem using a quantum-classical hybrid algorithm would verify a quantum advantage. On the other hand, the biggest disadvantage of this approach is that it is difficult to develop a quantum-classical hybrid algorithm that solves an LWE problem faster than classical algorithms and to estimate the number of qubits needed to do this.

A naive encoding strategy requires a tremendously large number of qubits to encode an open LWE challenge problem. The role of the LWE-reduction algorithm is to reduce an LWE problem to LWE-like problems with small moduli. Here, “LWE-like” means that the error distributions may have different standard deviations.

First, we introduced Algorithm 1, called LWE-reduction, which reduces an LWE problem to another LWE-like problem. Theorem 1 showed a pair consisting of a concrete solution and the associated error of the LWE-like problem. Algorithm 1 contains several bases and parameters to select. In general, depending on the setting of the bases and parameters, the LWE-like problem may have more than one solution. To make the solution unique, a standard setting of bases and parameters was introduced (Definition 1). Proposition 1 showed that the solution is expected to be unique when the bases and parameters are selected according to the standard setting. Theorem 2 showed that the solution of the LWE problem can be efficiently calculated from the solution of the LWE-like problem in the standard setting. Hence, it is sufficient to solve the LWE-like problem obtained using Algorithm 1 in the standard setting.

The smaller the modulus is, the easier it is to implement an LWE problem on a quantum computer. Unfortunately, when a modulus is decreased to an integer that is coprime to the modulus, the solution of the LWE problem cannot be easily calculated from the solution of the LWE-like problem obtained using Algorithm 1 in the standard setting. Since an LWE problem usually uses a prime modulus, Algorithm 1 in the standard setting cannot reduce the modulus. We thus used two stages to reduce the LWE problem. Algorithm 1 is run twice; the modulus of the LWE problem is increased to a power of two in the first stage and decreased to two in the second stage. Partial information about the solution of an LWE problem can be obtained from the solution of the reduced modulo-two LWE-like problem. To determine the solution of an LWE problem, the second stage is performed recursively. Algorithm 2 describes the recursive algorithm of the second stage. Theorem 3 showed that the solution of an LWE problem can be efficiently calculated from the solutions of modulo-two LWE-like problems under the assumption that the reduced modulo-two LWE-like problems are correctly solved.

A modulo-two LWE-like problem is represented as a MAX-SAT problem of a logical formula. A conversion from a MAX-SAT problem to an MIS problem is known^32^. The number of graph nodes of the MIS was evaluated using Theorem 4. An upper bound was depicted in Fig. 1. Although the number of qubits depends on the hardware, the minimum number of qubits necessary to solve an LWE problem is estimated as a few times the number of graph nodes, since the weights of the graph are small. For example, the minimum number of qubits necessary to solve a 40-dimensional LWE problem, which is the smallest-dimensional problem in the Darmstadt LWE challenge, was estimated at about a few times 40,000. Open LWE challenge problems will be within the reach of quantum computers in the near future.

We implemented the LWE-reduction algorithm. The prototype was written in the Wolfram language (Mathematica) and Python (SageMath). It successfully reduced the 40-dimensional LWE challenge problem with a relative error of 0.005 to a modulo-two LWE problem in the first recursive call. The graph of this problem was shown in Fig. 3. The prototype failed to reduce the 40-dimensional LWE challenge problem with a relative error of 0.035, which was an open LWE challenge problem as of June 21, 2021. (There were too many errors larger than one.) To successfully reduce this LWE problem, a more efficient lattice-reduction algorithm should be used.

In future research, a better lattice-reduction algorithm should be employed to perform the reduction. Replacing the lattice-reduction function used in the prototype with a state-of-the-art lattice-reduction algorithm would improve the prototype. Another research direction involves solving the reduced MIS problem (shown in Fig. 3) using a real quantum-inspired machine that has more than 100,000 qubits. The power of the quantum-classical hybrid algorithm will be made clearer by the experimental results of such machines.

## Data Availability

The datasets used and/or analyzed during the current study are available from the corresponding author on reasonable request.
